# Biomechanical Modeling of Pterygium Radiation Surgery: A Retrospective Case Study

**DOI:** 10.3390/s17061200

**Published:** 2017-05-24

**Authors:** Bojan Pajic, Daniel M. Aebersold, Andreas Eggspuehler, Frederik R. Theler, Harald P. Studer

**Affiliations:** 1 Eye Clinic Orasis, Swiss Eye Research Foundation, CH-5734 Reinach, Switzerland; bpajic@datacomm.ch; 2 Department of Physics, Faculty of Sciences, University of Novi Sad, 21000 Novi Sad, Serbia; 3 Division of Ophthalmology, Department of Clinical Neurosciences, Geneva University Hospitals, CH-1205 Geneva, Switzerland; 4 Faculty of Medicine of the Military Medical Academy, University of Defence, 11000 Belgrade, Serbia; 5 Department of Radiation Oncology, Inselspital, Bern University Hospital, University of Bern, CH-3010 Bern, Switzerland; daniel.aebersold@insel.ch; 6 Department of Neurology, Schulthess Klinik, CH-8008 Zuerich, Switzerland; a.eggspuehler@gmx.net; 7 Optimo Medical, CH-2503 Biel, Switzerland; frederik.theler@optimo-medical.com; 8 OCTlab, Department of Ophthalmology, University of Basel, CH-4001 Basel, Switzerland

**Keywords:** pterygium, radiation, cornea surgery, biomechanics, finite element modeling, simulation

## Abstract

Pterygium is a vascularized, invasive transformation on the anterior corneal surface that can be treated by Strontium-/Yttrium90 beta irradiation. Finite element modeling was used to analyze the biomechanical effects governing the treatment, and to help understand clinically observed changes in corneal astigmatism. Results suggested that irradiation-induced pulling forces on the anterior corneal surface can cause astigmatism, as well as central corneal flattening. Finite element modeling of corneal biomechanics closely predicted the postoperative corneal surface (astigmatism error −0.01D; central curvature error −0.16D), and can help in understanding beta irradiation treatment. Numerical simulations have the potential to preoperatively predict corneal shape and function changes, and help to improve corneal treatments.

## 1. Introduction

Pterygium, also called Surfer’s Eye, is a vascularized, invasive transformation growing on the anterior corneal surface, starting in the conjunctiva near the limbal region and with expanding towards the corneal center. The Bowmann’s membrane, underneath the epithelium, thereby serves as a controlling structure for the pterygium. Besides UV light, the following co-factors promoting the development of a pterygium have been reported in literature: chronic exposure to ultraviolet light in combination with hot and dry climate, chronic irritation by dust, and frequent exposure to wind [1]. The in-growth almost exclusively starts nasally (92%) [2], possibly because in that area the rays from the sun pass laterally through the cornea, intensifying the tissues’ exposure to UV light.

Even though co-factors are named, the main etiological reason for developing a pterygium is ultraviolet light exposure [3,4], as a recent mathematical model demonstrated that ultraviolet irradiation can lead to limbal stem cell dysfunction [5,6,7]. The fact that Fibroblast Growth Factor (FGF), Vascular Endothelial Growth Factor (VEGF), Transforming Growth Factor β (TGF β), and Stem Cell Factor (SCM) are increased in pterygial tissue [8,9,10], while IGFBP3 is decreased, further suggests that growth proliferation is not controlled in the same way as in tumor cells and, as a consequence, that pterygium is not neoplasia. Rather, it is a degenerative alteration [11], as VEGF leads to angiogenesis and SCM to the modulation of mast cells.

Various surgical treatments for pterygium have been suggested in the past and have been frequently employed in the field. However, depending on the technique, the recurrence rate of pterygium used to be relatively high, in a range from 35% to 68% [12,13,14]. More modern surgical procedures involving the implementation of antimetabolites, such as Mitomycin C, or the introduction of radiotherapy, decrease the recurrence rate down to a level of 1.7% to 12.5% [2,6,15,16,17,18]. Furthermore, therapy concepts such as pterygium excision with conjunctival autografts and subconjunctival amniotic membranes also reduce this rate down to a level of ~1% [19].

In their previous research [5,6,7,10,20], the authors of these papers showed that with the introduction of Strontium-/Yttrium-90 beta-irradiation as an exclusive, non-surgical treatment, no recurrences have occurred to date. Even though the result of beta-irradiation treatment is an inactive pterygium without vessels, the procedure may induce certain amounts of corneal astigmatism [5,6,7,10,20]. The authors hypothesized that the observed changes in cylinder value may stem from pulling forces placed the cornea by the retracting pterygium. Hence, it is of great importance to understand the underlying biomechanical connection between beta-irradiation and its pterygium reduction, as well as the induction of corneal astigmatism. The goal of this study is to develop a mathematical model to describe the governing biomechanical processes.

## 2. Materials and Methods

**Figure 1:**
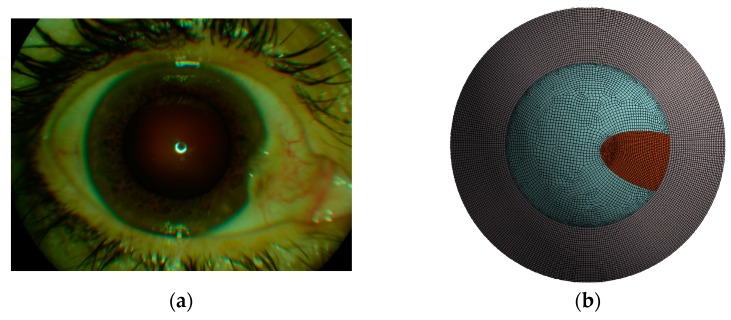
(**a**) Top-view image of the study subject with pterygium in-growth in the cornea; (**b**) Three-dimensional finite element model of the cornea and parts of the sclera, as seen from the top. The specific area (shown in red), representing where the pterygium pulls on the anterior corneal surface.

The right eye of a 56-year-old female subject, diagnosed with pterygium, was treated with Strontium-/Yttrium-90 irradiation treatment (see Figure 1a). Preoperative Pentacam (Oculus Optikgeräte GmbH, Wetzlar, Germany) measurements were taken to create a subject-specific finite element model. The model was numerically simulated using the Optimeyes software (Optimo Medical AG, Biel, Switzerland), employing an earlier published [21,22,23,24,25] constitutive material model. Optimeyes is a comprehensive technology platform for the simulation and prediction of corneal shape and function changes, caused by mechanical interferences with the tissue. The software allowed us to create patient-specific finite element models from anterior segment tomography measurement data, compute initial stress-distribution, and run numerical simulations of cornea surgical treatments. Simulation results were then compared to the 25-month postoperative follow-up Pentacam measurements. Comparison included corneal shape and corneal function analysis.

### 2.1. Pterygium Surgery with Beta Irradiation

A convex plate with a diameter of 12 mm, attached to a pen-like holder, was used as a Strontium-/Yttrium-90 applicator. The radioactive substance was attached to the inner surface of the plate, and softly put onto the eye, well centered over the pterygium. To reduce irradiation exposure of the surrounding tissue, a surround of 0.002 mm stainless steel and 0.01 mm aluminum, fitted to the edge of the applicator plate, filtered the original Strontium-90 irradiation down to 3%, and the Yttrium-90 down to 60%. The irradiation application scheme was as follows: A dosage of 6 gray (1 Gy = 1 J/kg) of the ionizing radiation was applied twice a week, for three consecutive weeks. Hence, a total dose of 6 × 6 Gy was administered to the pterygium.

### 2.2. Constitutive Material Model

Biomechanically, corneal tissue is known for being nearly incompressible, having non-linear elastic characteristics, being highly inhomogeneous in-plane as well as over its thickness, and for revealing a high degree of anisotropy. In this work, we used a previously published biomechanical model [21,22,23,24,25] which used additive terms in a non-linear, hyper elastic strain energy function to describe the tissue characteristics. Generally speaking, strain-energy functions are derived from the laws of thermodynamics, and relate deformation (right-hand side of the equation) to deformation energy (left-hand side of the equation). The formulation used in this work was already available in the Optimeyes software, and is given as:

(1) $$\Psi =U+{\bar{\Psi }}_{m}\left[C_{10}\right]+\frac{1}{\pi }\int \Phi \cdot \left({\bar{\Psi }}_{f1}\left[\gamma_{m},\mu_{m}\right]+{\bar{\Psi }}_{f2}\left[\gamma_{k},\mu_{k}\right]\right)d\theta$$

where U is a penalty-term, preventing volume changes and therefore modeling the incompressibility of corneal tissue, ${\bar{\Psi }}_{m}$ is a non-linear adaptation of Hooke’s law—called neo-hookean—representing the tissue matrix with its proteoglycans and glycosaminoglycans, and ${\bar{\Psi }}_{f1}$ and ${\bar{\Psi }}_{f2}$ are anisotropic polynomial material functions [26] modeling the main collagen fibers and the cross-links, respectively. The probability distribution function Φ defines a realistic fiber distribution, as has been assessed through X-ray scattering by Aghamohammadzadeh et al. [27]. The distribution is defined in the model by assigning a weighting to each possible direction (0° to 180°) for any location in the model, and as a function of corneal depth. Material constants (Table 1) were determined using three sets of experimental data: one from button inflation experiments [28] and two (one superior-inferior strip, one superonasal-inferotemporal strip) from strip extensometry [29] experiments. The inverse finite element method was used to fit the above strain energy function to the experimental data from our earlier work [21].

### 2.3. Patient-Specific Radiation Surgery Simulation

**Figure 2:**
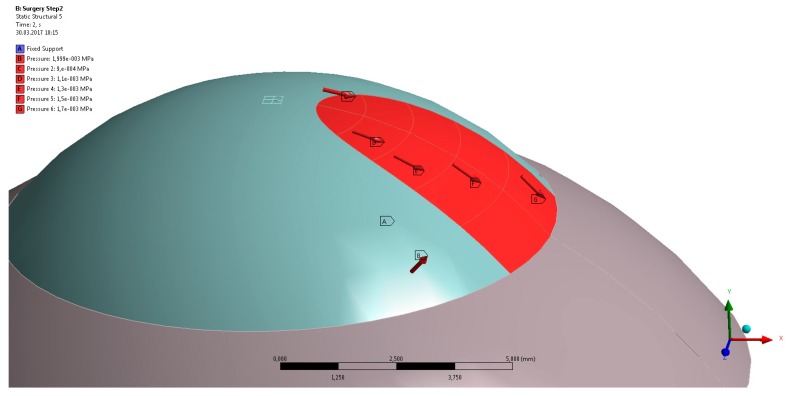
Three-dimensional finite element model of the cornea and the 4-mm scleral rim. The red area represents the specific part of the anterior surface of the cornea model where the tangential pulling forces were applied.

A patient-specific finite element model for the patient in the study was obtained with a three-step algorithm, available in the Optimeyes software: (i) The geometrical information obtained from spatial elevation data of the front and back surface of the patient’s cornea (acquired with the Scheimpflug tomography system Pentacam HR, Oculus Optikgeräte GmbH, Germany) was used to warp a spherical template cornea model to create a patient-specific finite element mesh containing 35,000 elements and over 44,000 nodes. (ii) The initial stress distribution in the model was then computed with an iterative approach [30,31]. (iii) Finally, the effects of the surgery were simulated. The anterior and posterior surfaces, computed by the finite-element (FE) model, were then compared to the postoperative surfaces to assess the accuracy and reliability of FE modeling. The details of the algorithm steps i–iii are described below:

(i) Mesh warping: In our earlier work [23], we showed that a model with patient-specific geometry of the human cornea can be obtained by warping a spherical finite element mesh such that its anterior and posterior surfaces match the respective surfaces of the tomography measurements. Thereby, the tomography surfaces are expressed as the coefficients obtained from Zernike expansion (up to the twelfth order, and over the central 8.0 mm optical zone of the cornea), and the inside mesh nodes proportionally follow the deformation of the respective surface nodes. This way, the template mesh was warped to match the patient’s cornea, without producing distorted elements (which is crucial for finite element analysis).
(ii) Calculation of initial stress distribution: Since the Pentacam Scheimpflug camera measures corneal geometry in vivo, whereby the corneal tissue is under mechanical stress, the shape in the absence of acting forces is a priori not known. An iterative approach to calculate the initial stress distribution in the model, as was previously published [30,31], was employed in this study.
(iii) Surgery simulation: A specific, three-dimensional, finite element model was created in the finite element software package ANSYS 17.1 (ANSYS Inc., Canonsburg, PA, USA). The model represents the full cornea, plus a 4-mm wide rim of scleral tissue. The model was fixed at the edge of the scleral rim, and a pressure of 15 mmHg on the models inside represented the intraocular pressure. The anterior surface of the model cornea is prepared such that a specific part exactly corresponds to the shape and position of the subject’s pterygium (see Figure 1b). Pressure was applied to that specific part, modeling the pulling forces of the retracting pterygium, tangentially to the corneal surface and towards the limbus (see Figure 2). This simulation approach reproduces the pulling effects placed onto the corneal surface when radiation-induced tissue shrinking in pterygium tissue occurs. The pterygium tissue itself was not modelled.

The anterior and posterior surface final geometry after finite element simulation were automatically imported, and then analyzed in the user interface of the Optimeyes software. The software uses an 8.0-mm region of interest for Zernike decomposition of the anterior and posterior corneal surface in the model, and the keratometric index *n* = 1.3375 for curvature calculation. Thereby, the sagittal curvature was calculated as $C_{S}=\left(n-1\right)/R$ , where R is the radius of the curvature, the normal distance between a surface point and the central axis of the cornea. From the sagittal curvatures, corneal astigmatism was calculated as the difference between the steep and flat simulated keratometry values over a central annulus of a 0.5- to 2.0-mm radius. Elevation data were calculated as the normal distance between the cornea and a reference surface, a best-fit sphere fitted over a central 8.0-mm diameter zone.

Corneal shape was compared by analyzing sagittal curvature maps of the anterior corneal surface. Color-coded curvature maps, provided by the Pentacam software as well as by the Optimeyes software, were used to calculate anterior corneal astigmatism, as well as central and paracentral corneal curvatures. Astigmatism was thereby calculated on an apex-centered annulus of 0.5 mm < r < 2.5 mm. Central corneal curvature is the average curvature on an apex-centered disk with a 2.0-mm radius. Paracentral corneal curvature is the average curvature on an apex-centered annulus of 2.0 mm < r < 3.5 mm. Corneal function was compared by analyzing anterior corneal wavefront indices over a central wavefront pupil with a 6-mm diameter.

Besides postoperative geometrical shape, the deformed finite element model also provides full-field biomechanical stress information for every simulation step as part of the software package. The average stress (and standard deviation) was calculated from the simulation within the central 3.0-mm zone.

## 3. Results

**Figure 3:**
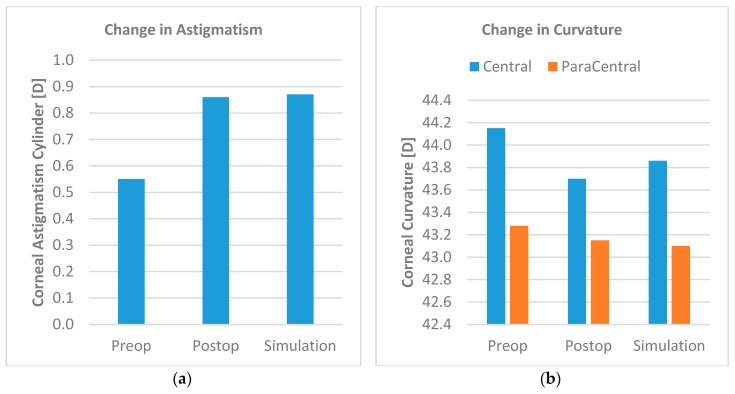
(**a**) Comparison of postoperative astigmatism cylinder and predicted cylinder. The simulation predicted the postoperative cylinder values very closely; (**b**) Comparison of postoperative central and paracentral curvatures and predicted curvature. Postoperative central and paracentral curvatures were well predicted by the simulation model.

Results from the patient-specific finite element simulations were compared to the actual clinical follow-up anterior segment tomography measurements. The simulation results showed a close match to the clinical data. While the simulation predicted an increase in astigmatism cylinder of +0.32D, in clinics, an increase of +0.31D was observed (see Figure 3a). The astigmatism axis did not change. The central corneal curvature decreased from 44.15D to 43.70D post-surgically. The simulation predicted a decrease to 43.86D. Furthermore, while the paracentral curvature decrease from 43.28D to 43.15D, the simulation predicted a decrease to 43.10D (see Figure 3b).

**Figure 4:**
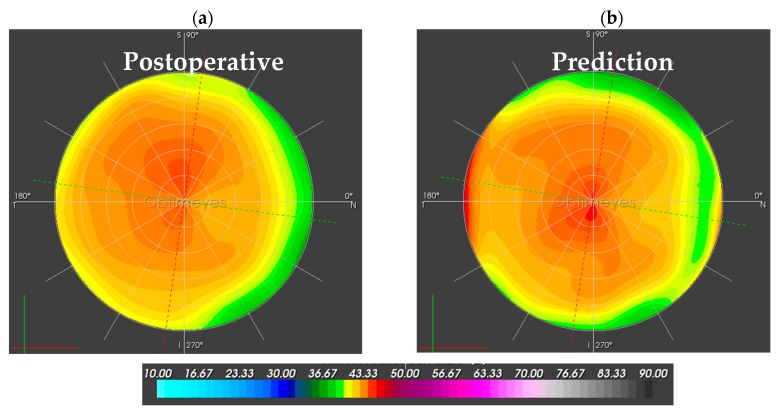
Sagittal anterior corneal curvature maps in Diopters [D] for the central 10.0-mm optical zone. (**a**) The left map is the post-surgical follow-up map, assessed by the Pentacam HR; (**b**) The right map shows the simulated prediction after the numerical simulation of pulling forces.

**Figure 5:**
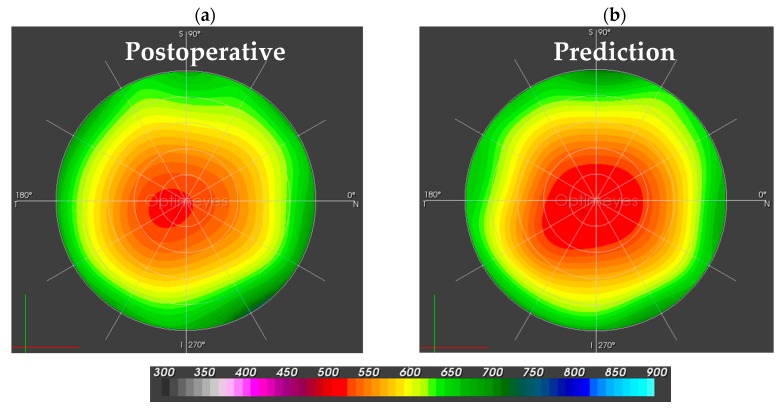
Corneal pachymetry maps, with a scale from 300 to 900 micrometers for the central 10.0-mm optical zone. (**a**) The left-hand side represents the postoperative pachymetry map; (**b**) The right-hand side depicts the simulated prediction of corneal thickness.

While Figure 4 compares the postoperative corneal sagittal curvature map to the predicted curvature map, Figure 5 shows the postoperative corneal pachymetry next to the predicted pachymetry map. Pachymetry slightly increased in clinics, as central corneal thickness went up from 512 to 528 micron, but remained stable in the simulation.

### 3.1. Corneal Function

**Figure 6:**
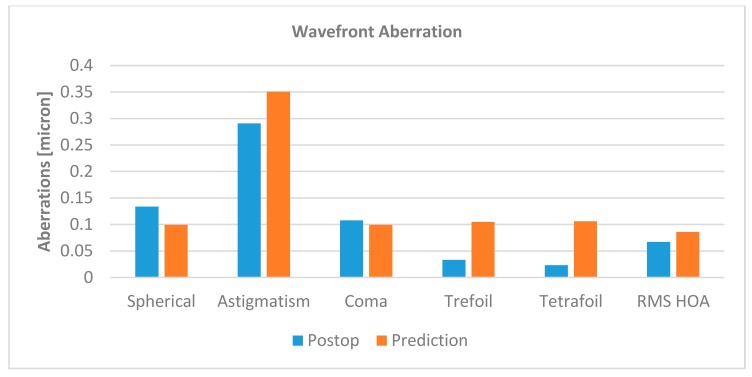
Zernike coefficients, given in micrometers, of spherical, astigmatic, coma, trefoil, tetrafoil, and root means square of higher order (RMS-HOA) wavefront aberrations. While the predicted values were comparable to postoperative wavefront coefficients for spherical (−26%), astigmatic (+21%), coma (−8%), and higher order aberrations (+28%), trefoil (+217%) and tetrafoil (+360%) were not well predicted.

Corneal function was analyzed by comparison of anterior corneal wavefront coefficients between the postsurgical and the simulated cornea. Figure 6 depicts spherical, astigmatic, coma, trefoil, and tetrafoil aberrations, as well as the root means square of higher order aberrations (Zernike order 4 and higher). Predicted spherical, astigmatic, and coma aberrations were close to the clinical follow-up measurements. The more irregular terms of trefoil and tetrafoil did not show a good match.

### 3.2. Corneal Biomechanics

**Figure 7:**
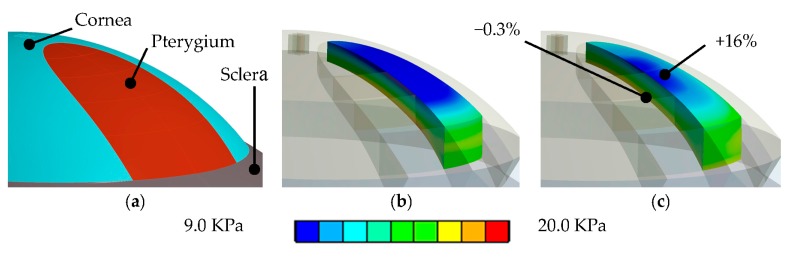
(**a**) Geometry of a cornea with pterygium. (**b**) Stress state before and (**c**) after applying the pulling force of the retracting pterygium. The color scale goes from blue (9 kPa) up to red (20 kPa). Therefore, blueish and greenish colors indicate low stress states, and orange and reddish colors indicate states of high stress. The simulation predicted an overall stress increase in corneal tissue under the pterygium of 2.4%.

Besides model deformation, finite element modeling allows for the calculation of mechanical stresses and strains. Stresses inside corneal tissue are computed as force over area, and are given in the unit kilo-pascal (kPa). Strain is the deformation relative to the initial dimension, and thus unit-less. Biomechanical simulation results showed an average stress increase in the tissue underneath the pterygium of 5% (from 13.7 kPa to 14.4 kPa). Strains in the area increased from 0.0110 to 0.0120 (4.8%). As Figure 7 shows, on the anterior corneal surface area of the pterygium, stresses increased by 16%, from 9.97 kPa to 11.60 kPa (on the same area on the posterior surface, stress change was negligible with −0.3%). Strains on the anterior corneal surface under the pterygium increased from 0.0083 to 0.0097, but did not change on the posterior surface under the same area (from 0.0147 to 0.0146).

## 4. Discussion and Conclusions

This work focused on numerical simulations of an earlier published beta-irradiation method for corneal pterygium treatment. The goal of the study was to better understand the relationship between Sr-90/Ytt-90 irradiation and clinically observed induction of corneal astigmatism, and to investigate the question of whether the retracting forces of a shrinking pterygium can be the cause of astigmatic changes. Furthermore, the model was intended to reveal the underlying biomechanical processes taking place during the treatment.

It has been shown in literature [6,7,10] that treatment with Sr-90/Ytt-90 irradiation only leads to the devascularization and reduction of the pterygium, without a single case of recurrence. Nevertheless, while corneal pachymetry remained stable after the treatment for all cases, changes in corneal astigmatism cylinder and axis were observed. It was hypothesized that the irradiation-induced retraction of the pterygium places pulling forces onto the anterior corneal surface, which as a consequence causes flattening along the central meridian of the pterygium, and ultimately leads to the induction of corneal astigmatism. Furthermore, clinical findings suggest that the amount of induced astigmatism depends on the preoperative extent of the pterygium. To the best of our knowledge, this is the first time that the mechanisms behind irradiation-induced astigmatic changes in the cornea are investigated with biomechanical simulations.

The results of biomechanical surgery simulation suggest that the clinically observed induction of corneal astigmatism after irradiation treatment might well be caused by pulling forces, exerted onto the anterior corneal surface by the retracting pterygium tissue. The simulation models, based on pre-treatment Pentacam examinations, reproduced pterygium treatment inside the computer and predicted the clinical outcome of a 25-month follow-up well, as compared to the acquired Pentacam data. Consequently, it appears likely that the biomechanical simulation model closely represents the clinical reality, and that it is biomechanical effects that cause the induction of corneal astigmatism. Since the applied pulling force of 30 Millinewton (mN) were chosen to predict postoperative astigmatism best, it remains to be proven clinically that this force corresponds to the actual forces created by pterygium retraction. 

Furthermore, simulation results suggest that, in addition to astigmatic changes, the cornea would also experience flattening. Interestingly, the predicted flattening effects in the central and paracentral cornea closely corresponded to the clinical results. On the other hand, predicted wavefront aberrations only partially matched with the clinical follow-up measurements. This might have to do with the fact that the simulation model was passed on the preoperative topography measurement and that because of the pterygium, the preoperative trefoil and tetrafoil aberrations might have been imprecisely assessed prior to the surgery. Still, important aberrations such as spherical, astigmatic, and coma aberrations were well predicted with by the simulation model. The fact that our clinical findings corresponded well with a theoretical model strongly supports the hypothesis that pterygium treatment by Sr-90/Ytt-90 irradiation can induce astigmatism as well as central and paracentral corneal flattening.

Even though the employed simulation model was patient-specific with respect to corneal shape, it has the limitation of assuming non-individualized biomechanical properties. Further limitations of the modeling are the assumed amount of pulling forces, the fact that the modeling only considered the anterior section of the eye and was working with an average intraocular pressure of 15 mmHg, and, most importantly, that modeling neglected potential radiation-induced tissue modifications and multi-physical effects. Nevertheless, the model still demonstrates that forces exerted on the cornea by the contracting pterygium may well be the root cause of induced corneal astigmatism as well as corneal flattening. Finite element modeling might help in the future to further understand the biomechanical effects of pterygium surgery, define improved treatment schemes, and reduce induced corneal shape changes.

## Figures and Tables

**Table 1:** Material coefficients, matching the age of the study subjects, that have been used in conjunction with the constitutive material model implementation. $C_{10}$ is the material constant of the neo-hookean hyper elastic material model for tissue matrix (proteoglycans and glycosaminoglycans, etc.), $\gamma_{m}$, $\mu_{m}$ are material constants of the polynomial material function $\Psi_{f1}$, introduced by Markert et al. [26], which model the main corneal collagen fibers, and $\gamma_{k}$, $\mu_{k}$ are material constants of the polynomial material function $\Psi_{f2}$, which model the collagen cross-links. Material coefficients were obtained from our earlier work [21].

$C_{10}\left[\mathrm{MPa}\right]$	$\gamma_{m}$	${𝛍}_{m}\left[\mathrm{MPa}\right]$	$\gamma_{k}$	${𝛍}_{k}\left[\mathrm{MPa}\right]$
0.06	0.13	24.0	0.08	95.0
